# Hypoxia-based classification and prognostic signature for clinical management of hepatocellular carcinoma

**DOI:** 10.1186/s12957-023-03090-x

**Published:** 2023-07-22

**Authors:** Ke Li, Yanfang Yang, Mingwei Ma, Suping Lu, Junjie Li

**Affiliations:** 1 Ruigu Medical Laboratory of Guangxi Medical University Co., LTD, Nanning, Guangxi China; 2 Guangxi Zhuoqiang Technology Co. LTD, Nanning, Guangxi China; 3 The First Affiliated Hospital of Guangxi Medical University, Nanning, Guangxi China; 4 Foresea Life Insurance Nanning Hospital, Nanning, Guangxi China

**Keywords:** Hypoxia, Hepatocellular carcinoma, RNF145, Prognosis, Genetic mutation, Immunotherapy, Heterogeneity

## Abstract

**Objective:**

Intratumoral hypoxia is an essential feature of hepatocellular carcinoma (HCC). Herein, we investigated the hypoxia-based heterogeneity and relevant clinical implication in HCC.

**Methods:**

Three HCC cohorts: TCGA-LIHC, LICA-FR, and LIRI-JP were retrospectively gathered. Consensus clustering analysis was utilized for hypoxia-based classification based upon transcriptome of hypoxia genes. Through LASSO algorithm, a hypoxia-relevant prognostic signature was built. Immunotherapeutic response was inferred through analyzing immune checkpoints, T cell inflamed score, TIDE score, and TMB score. RNF145 expression was measured in normoxic or hypoxic HCC cells. In RNF145-knockout cells, CCK-8, TUNEL, and scratch tests were implemented.

**Results:**

HCC patients were classified into two hypoxia subtypes, with more advanced stages and poorer prognosis in cluster2 than cluster1. The heterogeneity in tumor infiltrating immune cells and genetic mutation was found between subtypes. The hypoxia-relevant prognostic model was proposed, composed of ANLN, CBX2, DLGAP5, FBLN2, FTCD, HMOX1, IGLV1-44, IL33, LCAT, LPCAT1, MKI67, PFN2, RNF145, S100A9, and SPP1). It was predicted that high-risk patients presented worse prognosis with an independent and reliable manner. Based upon high expression of immune checkpoints (CD209, CTLA4, HAVCR2, SIRPA, TNFRSF18, TNFRSF4, and TNFRSF9), high T cell inflamed score, low TIDE score and high TMB score, high-risk patients might respond to immunotherapy. Experimental validation showed that RNF145 was upregulated in hypoxic HCC cells, RNF145 knockdown attenuated proliferation and migration, but aggravated apoptosis in HCC cells.

**Conclusion:**

Altogether, the hypoxia-based classification and prognostic signature might be useful for prognostication and guiding treatment of HCC.

**Supplementary Information:**

The online version contains supplementary material available at 10.1186/s12957-023-03090-x.

## Introduction

Hepatocellular carcinoma (HCC) arising from hepatocytes is one of the most prevalent and fatal causes of cancer-related death across the globe, with increasing incidence and mortality [1]. Although therapeutic advances have been recently made, including atezolizumab in combination with bevacizumab for unresectable HCC, survival outcomes remain poor, with five-year survival of only 15% owing to delayed diagnosis and limited response to existing treatment [2]. Liver transplantation enables to cure HCC in specific cases, but it is still a limited and resource-intensive therapeutic option, and most patients are ineligible for transplantation [3]. Therefore, it is critical to identify new approaches to predict the prognosis and treatment response for HCC patients. Due to the wide heterogeneity of risk factors and pathogenesis of HCC, existing predictive and prognostication approaches are still of limit.

Hypoxia, a drop in oxygen, is frequent in all solid cancers (including HCC) owing to abnormalities in the vascular system [4]. HCC is characterized by intratumoral hypoxia [5]. The median partial pressure of oxygen in HCC tissue is just 6 mmHg in comparison to 30 mmHg in normal liver tissue [6]. Hypoxia exerts widespread effects on biological behaviors and malignant phenotypes of HCC, involving metabolic reprogramming, stemness, invasion, metastases, angiogenesis, etc., thus synergistically contributing to progression and therapeutic resistance of HCC [7–9]. Immunotherapy has made great progress in HCC. Inadequate oxygen level may restrain successful T-cell activation or support T cell exhaustion, thus contributing to resistance to immune checkpoint inhibitors [10]. Identification of the hypoxia-relevant transcriptome profiling of tumors can infer responders and inform possible combination treatment. To solve existing problems, the present study proposed the hypoxia-based classification and prognostic signature for assisting prognostication and clinical management of HCC patients, which might facilitate individual-based treatment.

## Materials and methods

### Acquisition of human HCC cohorts

From the XenaBrowser (https://xenabrowser.net/datapages/), counts matrix and clinical data of the Cancer Genome Atlas (TCGA)-liver hepatocellular carcinoma (LIHC) were gathered. By use of CPM function of edgeR package [11], the counts data were converted into CPM. Genes with sample mean CPM value <10 were excluded. Two external transcriptome cohorts LICA-FR, and LIRI-JP were obtained from the International Cancer Genome Consortium (ICGC; https://icgc.org/).

### Consensus clustering analysis

Hypoxia genes were gathered from previous research. Based upon the transcriptome of hypoxia genes, consensus clustering was conducted on TCGA-LIHC samples utilizing ConsensusClusterPlus package [12]. The optimal number of clusters was determined with consensus matrix, consensus CDF, and tracking plot.

### Gene set enrichment analysis (GSEA)

According to gene expression as well as GO [13] and KEGG [14] databases, GSEA software was adopted for determining the difference in the specified gene sets between groups [15].

### Immune infiltration analysis

The fractions of tumor infiltrating immune cells were quantified in HCC tissues based upon transcriptome profiling by use of CIBERSORT deconvolution approach, with LM22 as the reference gene signatures [16].

### Genetic mutation analysis

Somatic variants in Mutation Annotation Format of HCC samples were gathered from TCGA database, which were analyzed utilizing Maftools package [17]. Significantly mutated genes identified by MutSigCV method were visualized into waterfall plots. Through implementing mafCompare function, differentially mutated genes were compared between groups via Fisher’s exact test. Genes mutated in mutually exclusive or co-occurring manners were identified through somaticInteractions function. Tumor mutational burden (TMB) was also computed in each sample.

### Differential expression analysis

Hypoxia-relevant genes were determined through comparing the gene expression between hypoxia subtypes based upon false discovery rate (FDR)<0.05 and |fold-change (FC)|>2.

### Prognostic signature establishment

Prognostic hypoxia-relevant genes with *p*<0.05 were screened via univariate-cox regression analysis, which were utilized for LASSO regression analysis using glmnet package [18]. Genes with coefficient≠0 were included for building a hypoxia-relevant prognostic signature. In accordance with 1:1, TCGA-LIHC samples were separated into the training and test datasets. By combining the transcript level and coefficient of each identified gene, hypoxia-relevant risk score was computed. Receiver operator characteristic curves (ROCs) were plotted for evaluating the predictive efficacy. Uni- and multivariate cox regression analyses were employed for determining whether the signature acted as an independent prognostic factor. The reproducibility of the signature was verified in LICA-FR, and LIRI-JP datasets.

### Drug sensitivity estimation

Based upon the transcriptome data and cgp2014 database, IC50 of small molecular agents was inferred by use of pRRophetic package [19]. The lower the IC50 value, the more sensitive to the agent.

### Immunotherapeutic response predictors

Immune checkpoints were gathered from previous research [20], and their expression was extracted. Cancer-testis antigens (CTA) were acquired from CTDatabae (http://www.cta. Incc.br/) [21]. Ayers et al. proposed a pan-cancer T cell inflamed score that enabled to define pre-existing cancer immunity and infer immunotherapeutic response. This study calculated T cell inflamed score as a weighted linear combination of the expression and coefficients of eighteen genes following the formula: T cell inflamed score = 0.008346 * CCL5 + 0.072293 * CD27 + 0.042853 * CD274 + (-0.0239) * CD276 + 0.031021 * CD8A + 0.151253 * CMKLR1 + 0.074135 * CXCL9 + 0.004313 * CXCR6 + 0.020091 * HLA-DQA1 + 0.058806 * HLA-DRB1 + 0.07175 * HLA-E + 0.060679 * IDO1 + 0.123895 * LAG3 + 0.075524 * NKG7 + 0.003734 * PDCD1LG2 + 0.032999 * PSMB10 + 0.250229 * STAT1 + 0.084767 * TIGIT [22]. TIDE computational approach was adopted to predict immunotherapeutic response based upon signatures of T cell dysfunction and exclusion [23].

### Cell culture and treatment

Hep3B and Huh7 (CTCC) were cultured in DMEM medium (L110KJ; Shanghai BasalMedia) supplemented with 10% fetal bovine serum (SV30087.02; Hyclone), which were planted onto a 6-well plate (5×10^5^ cells/well). After cultivating for 24 h in a 5% CO_2_ incubator at 37 °C, cells were exposed to 5% O_2_ hypoxia for 48 h.

### qRT-PCR

Cells were lysed with 1 mL Trizol (15596018; Invitrogen), and cDNA was synthesized by use of the first-strand cDNA synthesis kit. qRT-PCR was then conducted on ABI 7500 RT-PCR instrument (ABI), and analyzed via ABI Prism 7500 SDS Software. The sequences of primers included: Human RNF145 F: GACTGCTCTGCTCCTCTA, R: ACCACCAACTGACCTATT; human GAPDH F: GGAGCGAGATCCCTCCAAAAT, R: GGCTGTTGTCATACTTCTCATGG.

### Western blot

Cells were lysed with 100 μL RIPA reagent plus 1 μL PMSF on the ice for 30 min. At 12000 rpm centrifugation at 4 °C for 20 min, supernatant was gathered and protein was quantified with BCA kit (BL521A; Biosharp). 30 μg protein was loaded onto each well. After electrophoresis, protein samples were transferred onto PVDF membrane (HATF00010; Millipore). The membrane was sealed with 5% BSA (A9647; BIOSHARP) at room temperature for 1 h, followed by incubation with RNF145 (1:500; 24524-1-AP; Proteintech) or GAPDH (10494-1-AP; Proteintech) antibody at 4 °C. Next, incubation with secondary antibody was conducted at 37 °C for 1 h. The ECL luminescent solution (ECL-0011; Beijing Dingguo) was covered with the membrane. Exposure and image acquisition were conducted on ECL luminometer.

### Transfection

Cells were seeded onto a 96-well plate (1×10^4^ cells/well) and cultivated in an incubator of 37 °C, 5% CO_2_ for 24 h. 3 pmol siRNA of RNF145 (si-RNF145; Genepharma) or negative control (si-NC; Genepharma) as well as 0.3 μL Lipofectamine 2000 (52887; Invitrogen) was separately diluted with 5 μL culture medium, mixed, and incubated for 10 min at room temperature. The two were then mixed and incubated for 15 min at room temperature, which was added each well. After 48 h, transfection effect was evaluated through quantitative real-time polymerase chain reaction (qRT-PCR).

### CCK-8

Transfected cells were seeded onto a 96-well plate (1×10^4^ cells/well). After 48 h, 10 μL 5 mg/mL CCK-8 (C0038; Beyotime) was added to each well and incubated in a 5% CO_2_ incubator for 1 h at 37 °C away from the light. Optical density value at 450 nm was measured by use of microplate reader.

### TUNEL staining

Transfected cells were planted onto a 96-well plate (1×10^4^ cells/well). The samples were incubated with 50 μL TUNEL detection reagent (C1089; Beyotime) for 24 h at 37 °C in the dark.

### Scratch assay

A marker pen was utilized to evenly draw a line on the back of 3.5 cm dish. 5×10^5^ cells were seeded in 3.5-cm dishes and cultivated overnight. When the cell density reached ~90%, the scratch was made with 200 μL spear. The scratched cells were removed with PBS. Images were photographed at 0 h and 24 h under a microscope.

### Statistical analysis

Comparison between two groups was measured via Student’s t-test or Wilcoxon test. Multi-group comparison was implemented utilizing one- or two-way analysis of variance. Kaplan-Meier (K-M) curves of overall survival (OS), disease-free survival (DFS), disease-specific survival (DSS), and progression-free survival (PFS) were plotted, with log-rank test for estimating survival difference. All statistical analysis was achieved by use of R packages and Graph Prism 9.0.1. Pearson or Spearman test was utilized for investigating the correlation between variables *P*<0.05 indicated statistical significance.

## Results

### Establishment of two hypoxia subtypes with diverse clinicopathologic features and prognosis in HCC

**Fig. 1:**
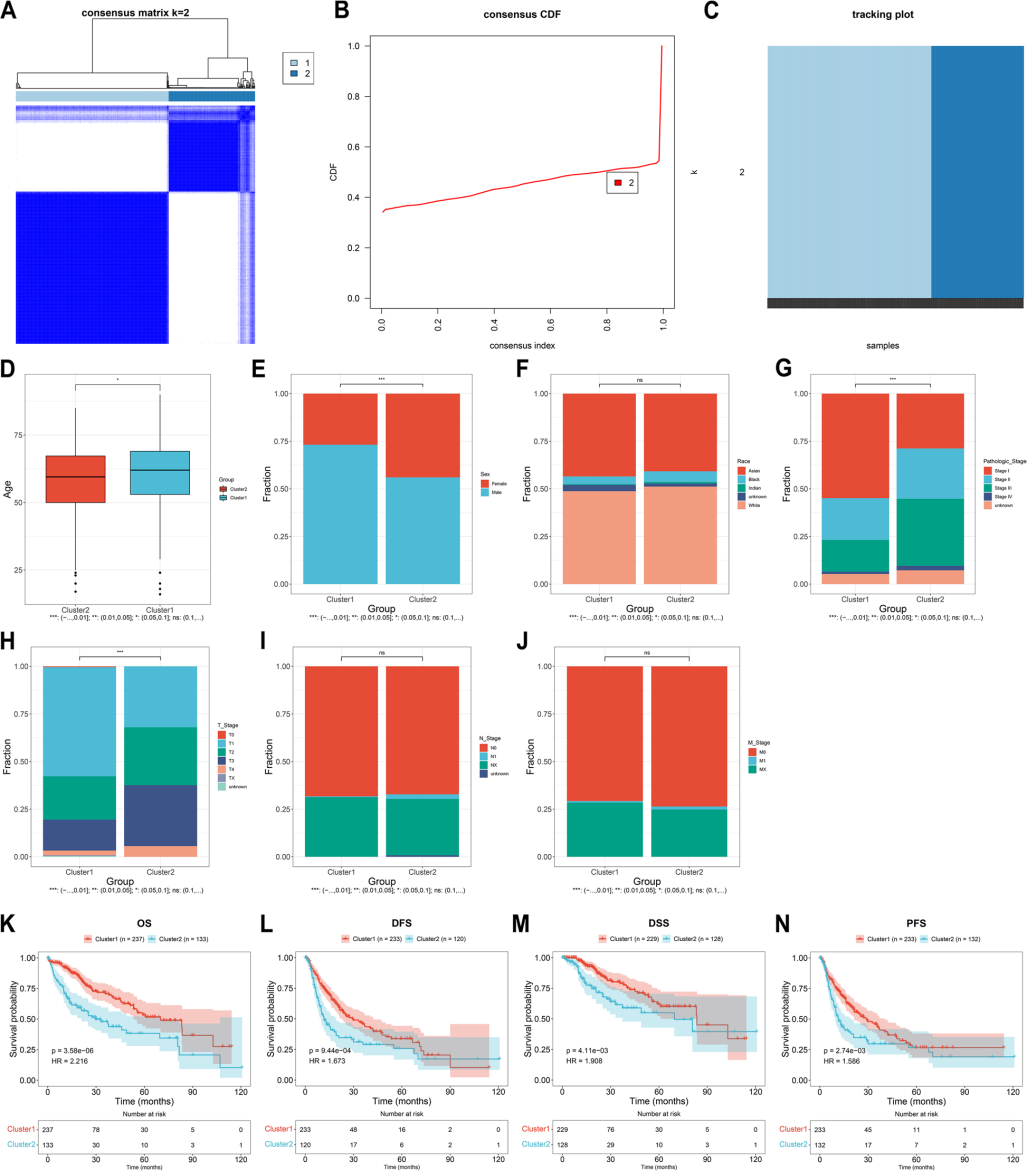
Establishment of two hypoxia subtypes with diverse clinicopathologic features and prognosis in TCGA-LIHC dataset. **A**-**C** Consensus matrix, consensus CDF, and tracking plot at k=2 based upon the transcript levels of hypoxia genes. **D**-**J** Distribution of age, sex, race, pathologic stage, T, N, M stage. (K-N) K-M curves of OS, DFS, DSS, and PFS outcomes between cluster1 and cluster2. *P<0.05; ***p<0.001; ns: no significance

According to the hypoxic transcriptomes, we classified TCGA-LIHC samples as two hypoxia subtypes by the use of consensus clustering approach (Fig. 1A-C). Each hypoxia subtype possessed unique clinicopathologic features. In comparison to cluster2, cluster1 displayed older age, higher proportion of males, lower proportions of advanced pathologic stage and T stage, without difference in race, N, and M stage (Fig. 1D-J). Survival difference was also observed between subtypes. Cluster2 presented worse OS, DFS, DSS and PFS outcomes relative to cluster1 (Fig. 1K-N).

### Hypoxia subtypes with distinct signaling pathways, immune cell infiltration, PD-L1 expression, genetic mutations

**Fig. 2:**
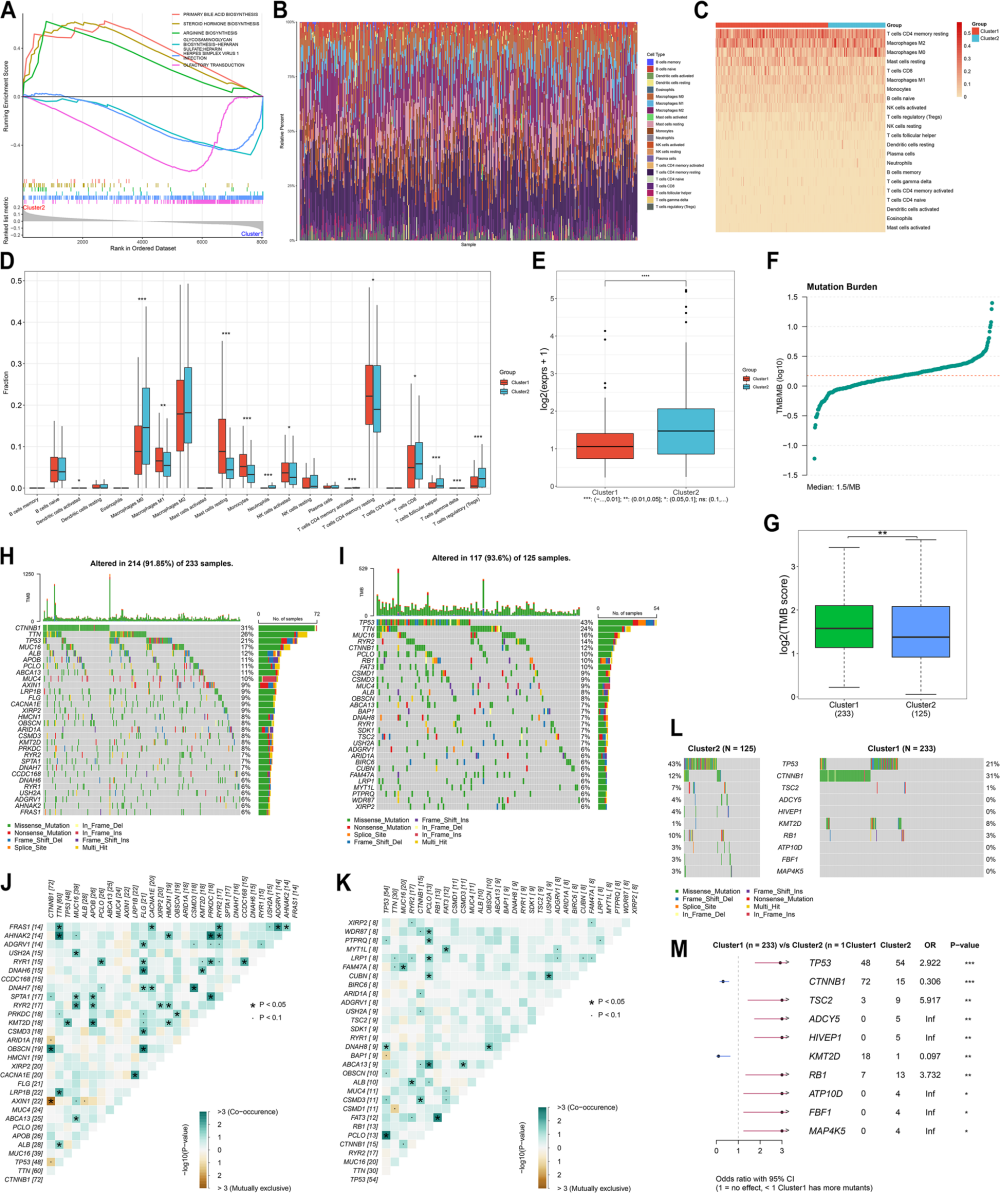
Two hypoxia subtypes characterized by distinct signaling pathways, immune cell infiltration, PD-L1 expression, and genetic mutation across TCGA-LIHC samples. **A** GSEA of the major KEGG pathways with different enrichment in two hypoxia subtypes. **B** Landscape of the relative infiltration of immune cells across TCGA-LIHC tissues. **C**, **D** Comparison of immune cell infiltration between hypoxia subtypes. **E** Difference in PD-L1 expression between cluster1 and cluster2. **F** Distribution of TMB score across TCGA-LIHC samples. **G** Difference in TMB score between subtypes. **H**, I waterfall plots of the somatic landscape in two hypoxia subtypes cluster1 and cluster2. Genes are ranked according to mutational frequency. Side bar plot displays the percentage of mutated samples. **J**, **K** Mutually exclusive and co-occurring mutated genes in cluster1 and cluster2. Green denotes co-occurrence and brown denotes exclusivity. **L** Comparison of mutated genes between cluster1 and cluster2. **M** Differentially mutated genes between subtypes. Bar indicates 95% confidence interval of odd ratio (OR). The right table displays the number of samples in two subtypes with the mutation in the indicated gene. *P<0.05; **p<0.01; ***p<0.001; ****p<0.0001

The heterogeneity in signaling pathways was found in two hypoxia subtypes, with the significant enrichment of primary bile acid biosynthesis, steroid hormone biosynthesis, and arginine biosynthesis in cluster2 as well as the significant enrichment of biosynthesis-heparan sulfate, heparin herpes’s simplex virus 1, infection, and olfactory transduction in cluster1 (Fig. 2A). The fractions of immune cells were inferred across TCGA-LIHC samples through CIBERSORT (Fig. 2B). In comparison to cluster1, M0 macrophages, neutrophils, T cells CD8, T cells follicular helper, and T cells regulatory (Tregs) presented the higher fractions in cluster2, with the lower fractions of M1 macrophages, mast cells resting, monocytes, NK cells activated, and T cells CD4 memory resting (Fig. 2C, D). In addition, higher PD-L1 expression was measured in cluster2 relative to cluster1 (Fig. 2E), indicating that patients in cluster2 might respond to anti-PD-L1 therapy. TMB reflects cancer mutation quantity [24]. Here, we calculated TMB score in TCGA-LIHC samples (Fig. 2F). Cluster1 exhibited higher TMB score relative to cluster2 (Fig. 2G). Two hypoxia subtypes had the heterogeneity in genetic mutation (Fig. 2H, I). Higher mutually co-occurring mutated gene pairs were found in cluster1 versus cluster2 (Fig. 2J, K). After comparison, cluster1 displayed the higher frequency of CTNNB1, and KMT2D, with the higher frequency of TP53, TSC2, ADCY5, HIVEP1, RB1, ATP10D, FBF1, and MAP 4K5 in cluster2 (Fig. 2L, M).

### Identification of hypoxia-relevant genes

**Fig. 3:**
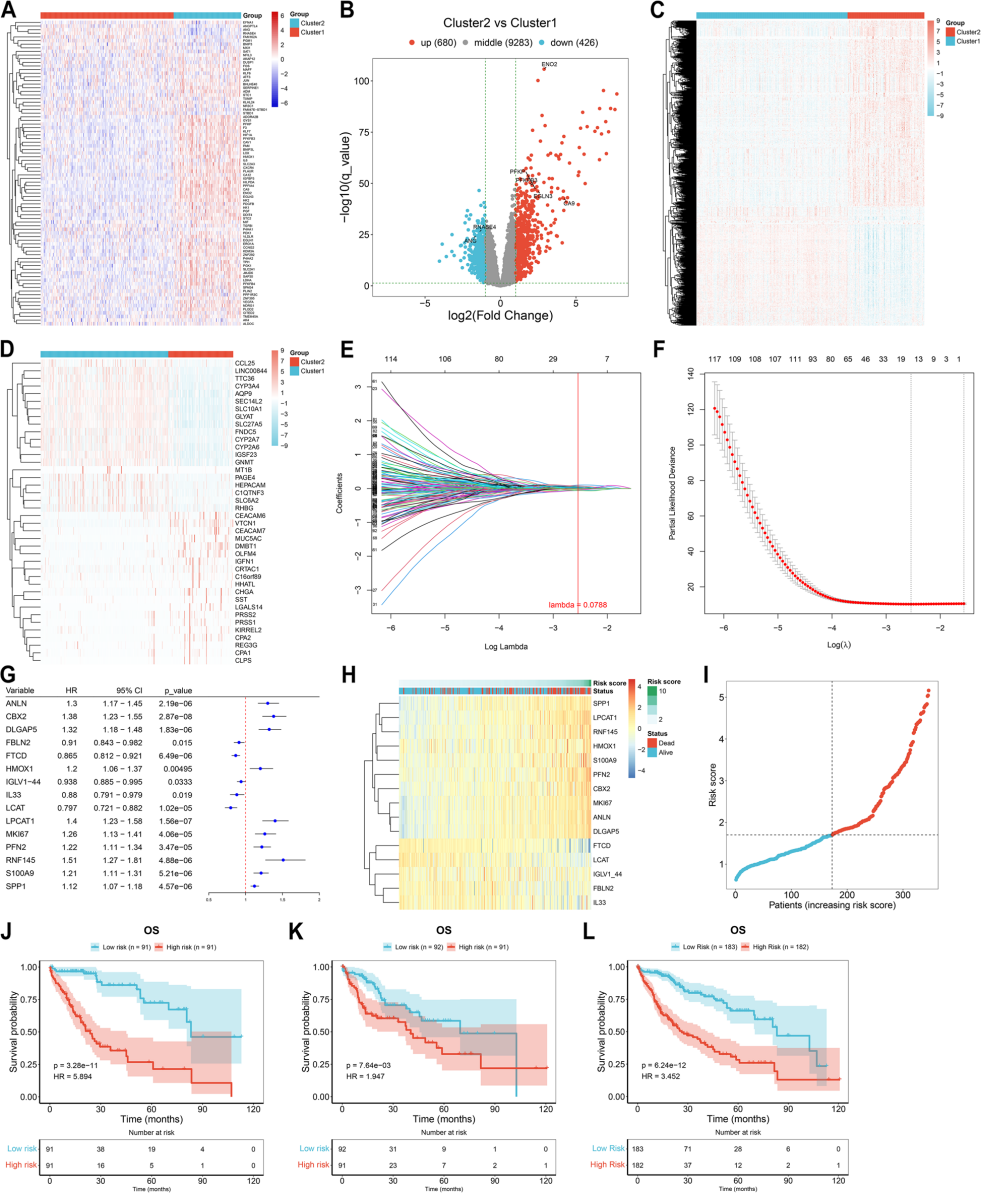
Screening hypoxia-relevant genes and definition of a hypoxia-relevant prognostic signature in TCGA-LIHC dataset. **A** Expression difference of hypoxia genes in cluster1 versus cluster2. **B**, **C** Identifying hypoxia-relevant genes with differential expression between cluster1 and cluster2. **D** The top 20 hypoxia-relevant genes in cluster1 and cluster2. **E** LASSO coefficient results based upon prognostic hypoxia-relevant genes. **F** Ten-fold cross-validation. **G** Univariate-cox regression results of genes screened by LASSO. **H** Expression of identified genes in hypoxia-relevant risk score. **I** Distribution of hypoxia-relevant risk score. **J**-**L** K**-**M curves of OS in high- versus low-risk patients in the training, test and total datasets

Notably, the heterogeneity in transcript level of hypoxia genes was observed in two hypoxia subtypes (Fig. 3A). Most hypoxia genes presented the higher expression in cluster2 relative to cluster1. With FDR<0.05 and |FC|>2, 680 up-regulated genes and 426 down-regulated genes were identified in cluster1 versus cluster2, which were regarded as hypoxia-relevant genes (Fig. 3B, C). Especially, we displayed the top 20 hypoxia-relevant genes in each hypoxia subtype (Fig. 3D). Based upon univariate-cox regression results, 226 hypoxia-relevant genes acted as protective factors of HCC prognosis (Supplementary Table 1), with 258 genes as risk factors (Supplementary Table 2).

### Definition of a hypoxia-relevant prognostic signature

Prognostic hypoxia-relevant genes were included in LASSO analysis. Genes with regression coefficients not equal to 0 (including ANLN, CBX2, DLGAP5, FBLN2, FTCD, HMOX1, IGLV1-44, IL33, LCAT, LPCAT1, MKI67, PFN2, RNF145, S100A9, and SPP1) were selected for constructing a hypoxia-relevant prognostic signature under the minimum λ = 0.0788 (Fig. 3E, F). Univariate-cox regression analysis revealed the prognostic implication of the identified genes (Fig. 3G). Through combining coefficient and transcript level of the identified genes, hypoxia-relevant risk score was computed with the formula of risk score = 0.01173193 * ANLN + 0.026334473 * CBX2 + 0.06567423 * DLGAP5 + (-0.027023234) * FBLN2 + (-0.0595385) * FTCD + 0.006369158 * HMOX1 + (-0.048110046) * IGLV1-44 + (-0.059442624) * IL33 + (-0.010601843) * LCAT + 0.099289921 * LPCAT1 + 0.01552718 * MKI67 + 0.075613467 * PFN2 + 0.014509309 * RNF145 + 0.04114437 * S100A9 + 0.029302715 * SPP1. We also observed the differential expression of the identified genes across patients (Fig. 3H). Based upon the median risk score, the present study stratified HCC cases into low- and high-risk groups (Fig. 3I). With the ratio of 1:1, TCGA-LIHC samples were equally divided into the training and test datasets. In the training dataset, low-risk group presented the better OS outcomes relative to high-risk group (Fig. 3J). The OS difference was proven in the test and entire datasets (Fig. 3K, L).

### Hypoxia-relevant risk score correlates to clinicopathologic features of HCC

**Fig. 4:**
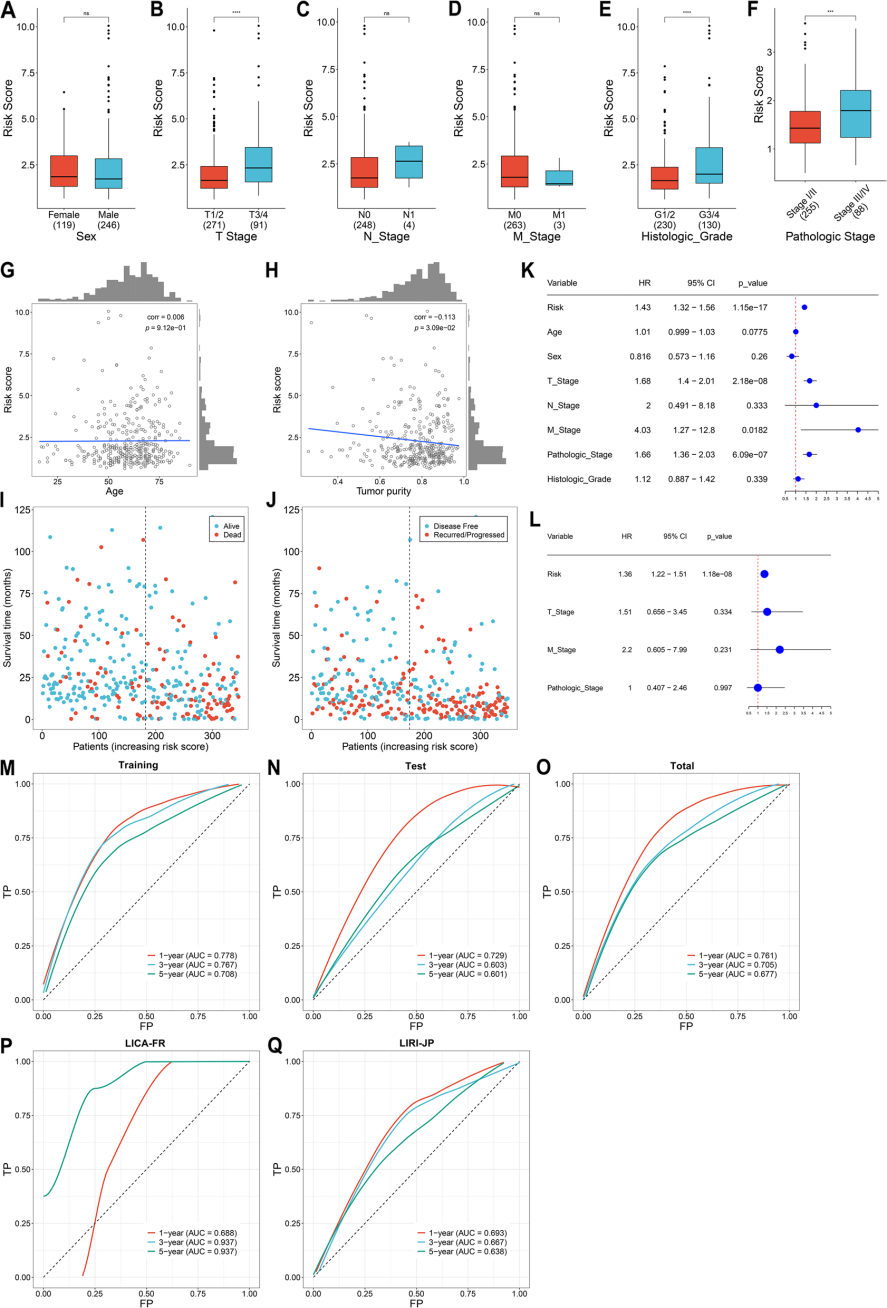
Hypoxia-relevant risk score correlates to clinicopathologic features and independently and reliably predicts HCC prognosis. **A**-**F** Comparison of risk score between different clinicopathologic factors: gender, T, N, M stage, histologic grade, and pathologic stage in TCGA-LIHC dataset. **G**, **H** Relationship of risk score with age, and tumor purity across TCGA-LIHC patients. **I** Distribution of alive and dead status in low- and high-risk patients. **J** Distribution of disease free and recurred/progressed status in two groups. **K**, **L** Uni- and multivariate cox regression results on risk score and clinicopathologic factors with TCGA-LIHC prognosis. **M**-**O** ROCs at one-, three- and five-year survival in the training, test and total datasets. **P**, **Q** ROCs in the LICA-FR, and LIRI-JP datasets. ***P<0.001; ****p<0.0001; ns: no significance

Next, we compared hypoxia-relevant risk score between different clinicopathologic factors: gender, T, N, M stage, histologic grade, and pathologic stage. As a result, higher risk score was observed in T3/4 versus T1/2, G3/4 versus G1/2, stage III/IV versus I/II, without significant difference between distinct gender, N stage, and M stage (Fig. 4A-F). In addition, there was no significant relationship of hypoxia-relevant risk score with age (Fig. 4G). As illustrated in Fig. 4H, hypoxia-relevant risk score was negatively linked with tumor purity. More dead and recurred/progressed cases were also found in high-risk group (Fig. 4I, J).

### Hypoxia-relevant risk score independently and reliably predicts HCC prognosis

Univariate-cox regression results demonstrated that hypoxia-relevant risk score, T stage, M stage, and pathologic stage were significantly correlated to HCC prognosis (Fig. 4K). Based upon multivariate-cox regression results, hypoxia-relevant risk score served as an independent risk factor of HCC prognosis (Fig. 4L). ROCs were plotted to evaluate the efficacy of hypoxia-relevant risk score in prognosis prediction. In the training dataset, the AUC values at one-, three- and five-year survival were all >0.7, demonstrating the high sensitivity and specificity in predicting prognostic outcomes (Fig. 4M). The well prediction efficacy was proven in the test, and total datasets (Fig. 4N, O). To verify the reproducibility, LICA-FR, and LIRI-JP datasets were adopted. Consequently, hypoxia-relevant risk score reliably predicted patient prognosis (Fig. 4P, Q).

### Molecular mechanisms underlying hypoxia-relevant risk score

**Fig. 5:**
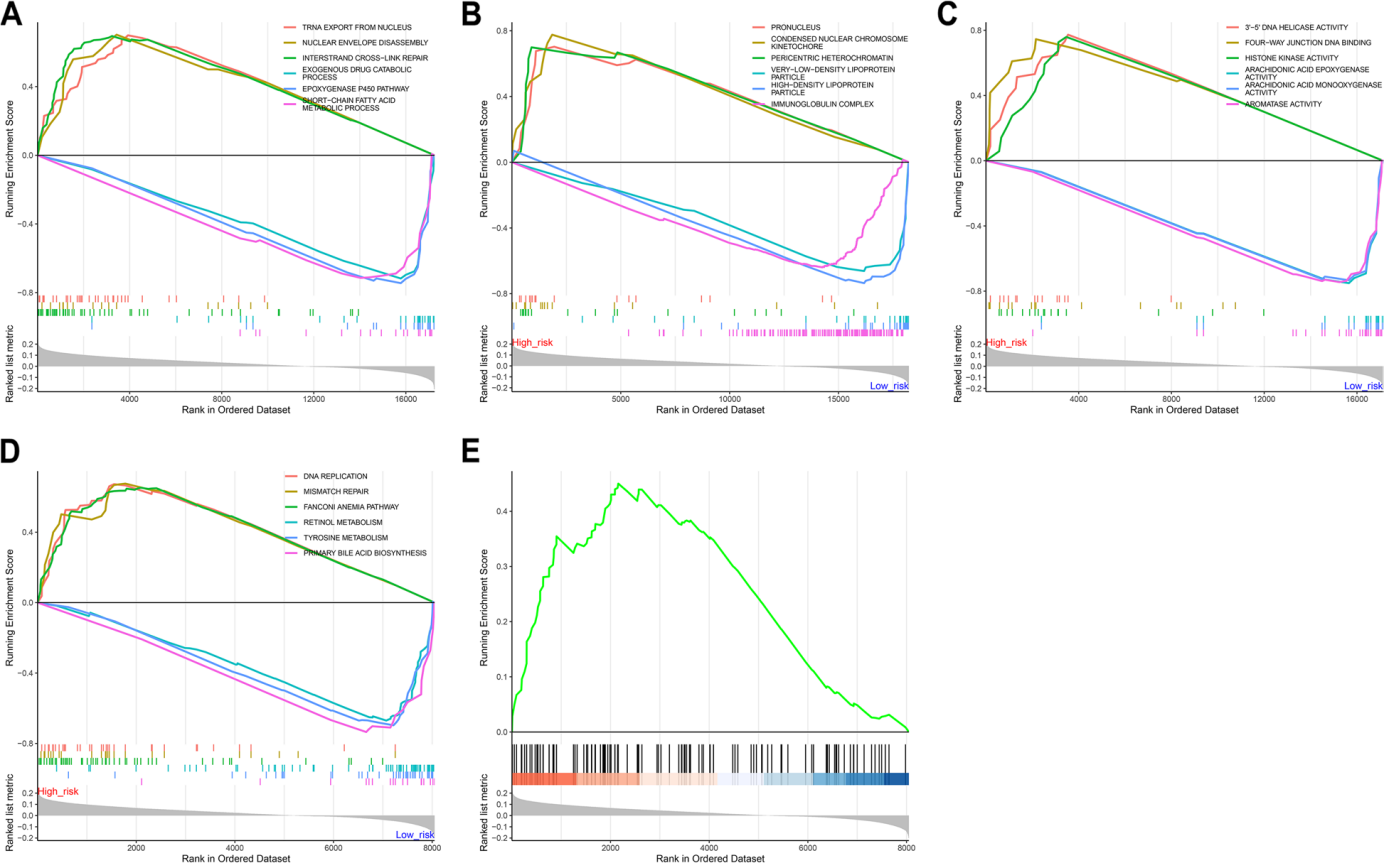
Molecular mechanisms underlying hypoxia-relevant risk score in TCGA-LIHC dataset. **A**-**D** GSEA showing the biological processes, cellular components, molecular functions, and KEGG pathways with different enrichment between low- and high-risk groups. **E** HIF signaling pathway with significant difference between groups

For biological process, tRNA export nucleus, nuclear envelope disassembly, and interstrand cross-link repair were significantly enriched in high-risk group, with significant enrichment of exogenous drug catabolic process, epoxygenase P450 pathway and short-chain fatty acid metabolic process in low-risk group (Fig. 5A). For cellular component, pronucleus, condensed nuclear chromosome kinetochore presented the significant enrichment in high-risk samples, while very-low-density lipoprotein particle, high-density lipoprotein particle, and immunoglobulin complex were notably enriched in low-risk samples (Fig. 5B). For molecular function, high-risk group had the prominent enrichment by 3’-5’ DNA helicase activity, four-way junction DNA binding, and histone kinase activity (Fig. 5C). Meanwhile, low-risk group exhibited the significant enrichment by arachidonic acid epoxygenase activity, arachidonic acid monooxygenase activity and aromatase activity. KEGG pathway enrichment results showed that DNA replication, mismatch repair, Fanconi anemia pathway were significantly enriched in high-risk group, with significant enrichment of retinol metabolism, tyrosine metabolism, and primary bile acid biosynthesis in low-risk group (Fig. 5D). Especially, we focused on HIF signaling pathway with significant enrichment in high-risk group (Fig. 5E).

### Prediction of possible small molecular agents based upon hypoxia-relevant risk score

**Fig. 6:**
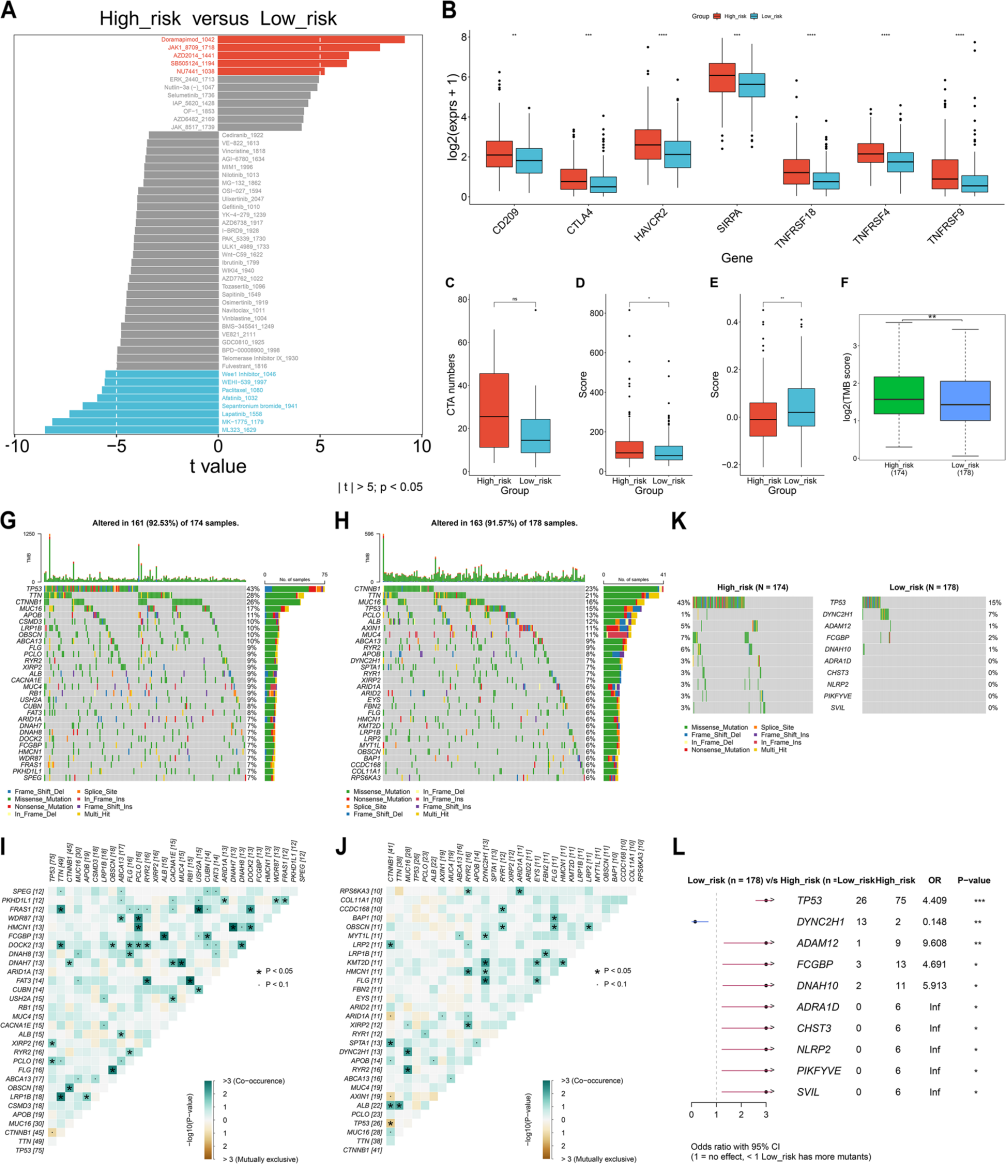
Heterogeneity in drug sensitivity, immunotherapeutic response and genetic mutation between low- and high-risk HCC patients in TCGA-LIHC cohort. **A** Difference in IC50 of small molecular agents between low- and high-risk patients. **B** Immune checkpoints with significant difference between groups. **C**-**F** Comparison of CTA, T cell inflamed score, TIDE score and TMB between groups. **G** Waterfall plot illustrating the major mutated genes in high-risk group. **H** Waterfall plot exhibiting the major mutated genes in low-risk group. **I** Interactions between mutated genes across high-risk patients. **J** Interactions between mutated genes across low-risk patients. **K**, **L** Comparison of the mutation frequency between low- and high-risk groups. *p<0.05; **p<0.01; ***p<0.001; ****p<0.0001; ns: no significance

Small molecular agents including Doramapimod_1042, JAK1_8709_1718, AZD2014_1441, SB505124_1194, NU7441_1038, ML323_1629, MK-1775_1179, Lapatinib_1558, Sepantronium bromide_1941, Afatinib_1032, Paclitaxel_1080, WEHI-539_1997, and Wee1 Inhibitor_1046 exhibited the notable difference in IC50 between low- and high-risk HCC patients (Fig. 6A), which might potentially treat HCC.

### Hypoxia-relevant risk score predicts the response to immunotherapy

Among known immune checkpoints, CD209, CTLA4, HAVCR2, SIRPA, TNFRSF18, TNFRSF4, and TNFRSF9 presented the higher transcript level in high- relative to low-risk group (Fig. 6B). CTA are tumor antigens that experience dysregulated expression in tumor and malignant cells. However, no remarkable difference in CTA numbers was found between low- and high-risk patients (Fig. 6C). Higher T cell inflamed score and lower TIDE score were observed in high-risk group (Fig. 6D, E). Altogether, high-risk patients might benefit from immunotherapy.

### Heterogeneity in genetic mutation between low- and high-risk HCC patients

TMB was calculated to reflect cancer mutation quantity. The difference in TMB was observed between low- and high-risk patients, with higher TMB in high-risk patients (Fig. 6F). Overall, mutated samples occupied 92.53% in high-risk group, and 91.57% in low-risk group (Fig. 6G, H). Higher co-occurrence of mutated genes was found in high- relative to low-risk group (Fig. 6I, J). Specifically, TP53 (43% versus 15%), ADAM12 (5% versus 1%), FCGBP (7% versus 2%), DNAH10 (6% versus 1%), ADRA1D (3% versus 0%), CHST3 (3% versus 0%), NLRP2 (3% versus 0%), PIKFYVE (3% versus 0%), and SVIL (3% versus 0%) presented the higher mutated frequency in high- than low-risk patients (Fig. 6K, L). Oppositely, the mutated frequency of DYNC2H1 was lower in high- relative to low-risk group (1% versus 7%).

### RNF145 is up-regulated in hypoxic HCC cells

**Fig. 7:**
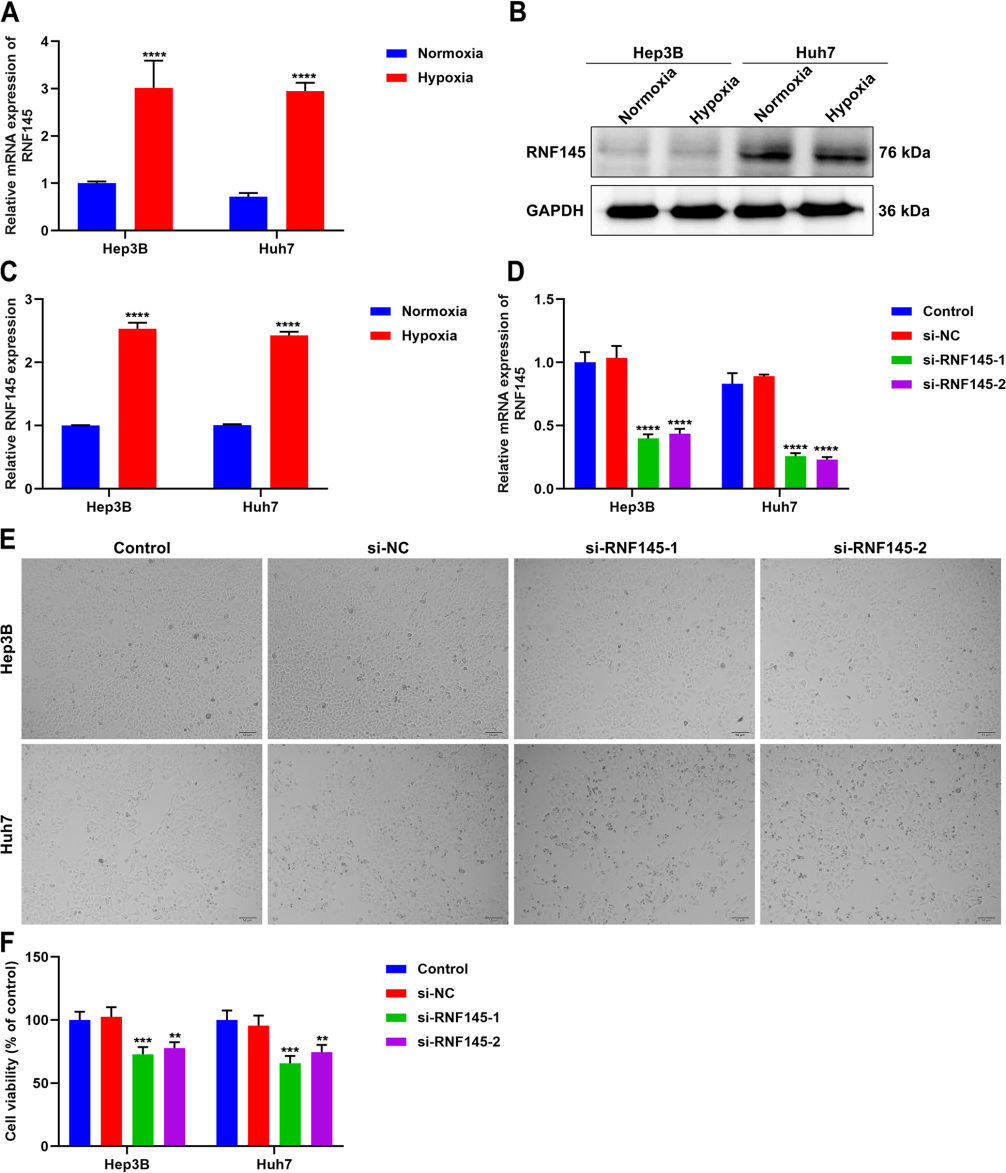
RNF145 is up-regulated in hypoxic HCC cells and its inhibition attenuates proliferation of HCC cells. **A** The mRNA level of RNF145 in normoxic or hypoxic Hep3B and Huh7 cells by use of qRT-PCR. **B**, **C** The protein level of RNF145 in normoxic or hypoxic HCC cells utilizing western blot. **D** Verification of the transfection effect of si-RNF145 in HCC cells at the mRNA level. **E** Representative photographs of HCC cells with si-RNF145 transfection. Bar, 50 μm. **F** The cell viability of RNF145-knockout HCC cells utilizing CCK-8. In comparison to si-NC group, **p<0.01; ***p<0.001; ****p<0.0001

Among hypoxia-relevant genes in the signature, the role of RNF145 in HCC remains unexplored. We observed that RNF145 expression was prominently up-regulated in hypoxic Hep3B and Huh7 cells at the RNA and protein levels (Fig. 7A-C), proving that RNF145 up-regulation might be affected by hypoxia in HCC.

### RNF145 inhibition attenuates proliferation of HCC cells

To measure whether RNF145 affects HCC progression, specific siRNAs of RNF145 were transfected into Hep3B and Huh7 cells. As illustrated in Fig. 7D, si-RNF145 transfection remarkably mitigated the expression of RNF145 in HCC cells. CCK-8 was conducted to detect the proliferation of HCC cells. Consequently, RNF145-knockout HCC cells exhibited impaired proliferative capacity (Fig. 7E, F), demonstrating that RNF145 might be essential for HCC cell growth.

### RNF145 suppression enhances apoptosis of HCC cells

**Fig. 8:**
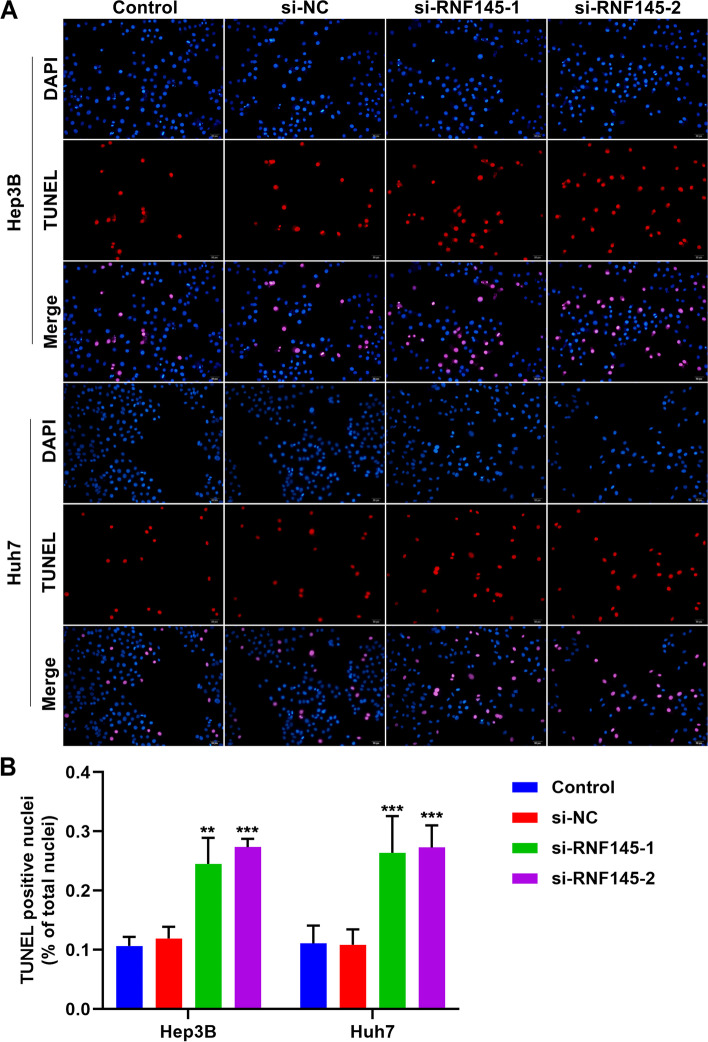
RNF145 suppression enhances apoptosis of HCC cells. **A** Representative photographs of TUNEL staining of si-RNF145 transfected Hep3B and Huh7 cells. Bar, 20 μm. **B** Percentage of TUNEL-positive nuclei in RNF145-knockout HCC cells. **P<0.01; ***p<0.001

TUNEL staining was implemented to measure the apoptosis of HCC cells. In RNF145-knockout Hep3B and Huh7 cells, percentage of TUNEL-positive nuclei was notably increased (Fig. 8A, B). This indicated that RNF145 suppression was capable of enhancing HCC cell apoptosis.

### RNF145 knockdown mitigates migration of HCC cells

**Fig. 9:**
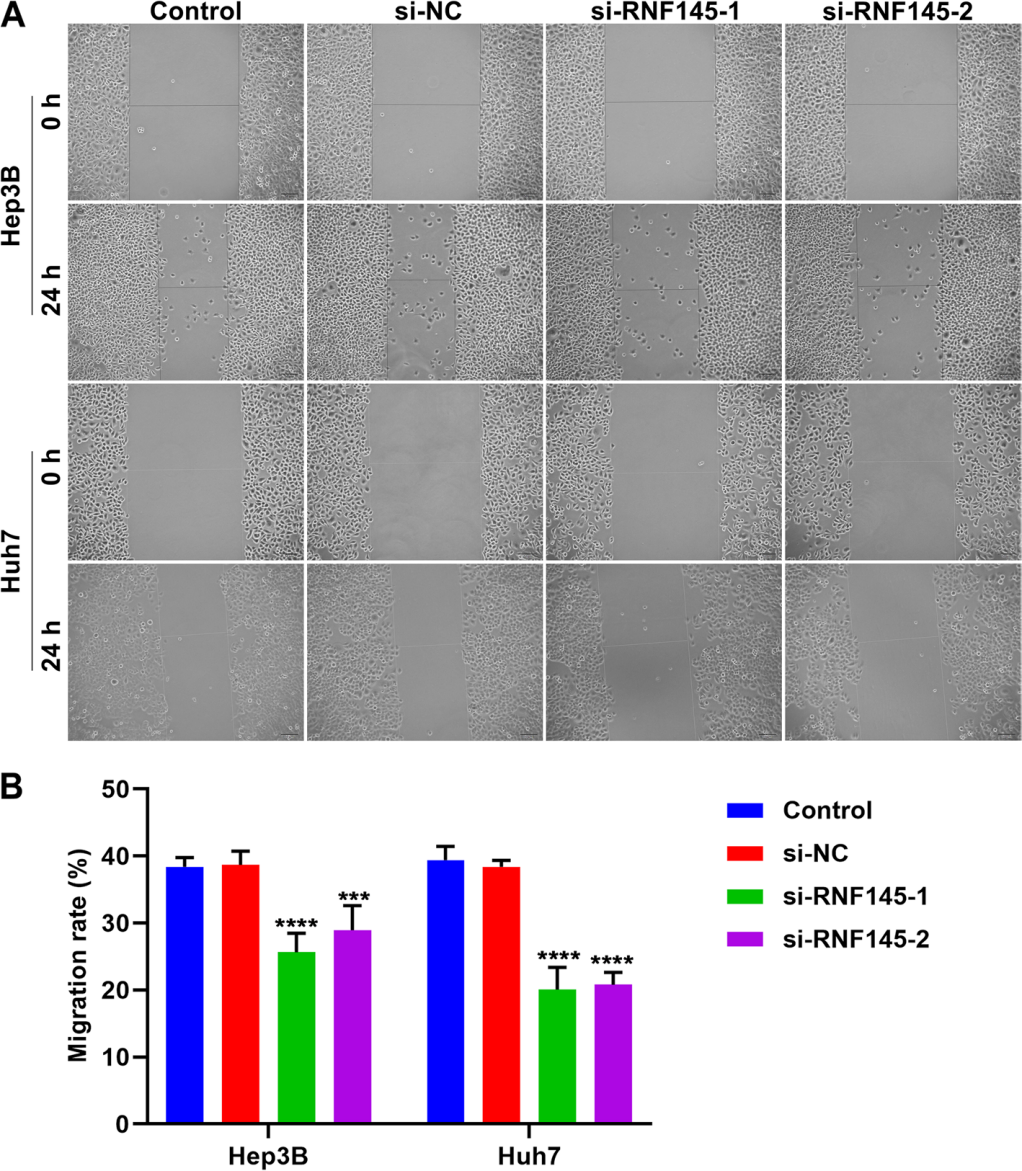
RNF145 knockdown mitigates migration of HCC cells. **A** Representative photographs of scratched Hep3B and Huh7 cells transfected with specific siRNAs targeting RNF145 at 0 h and 24 h. Bar, 50 μm. **B** Migration rate of HCC cells with RNF145 knockdown. Compared with si-NC group, ***p<0.001; ****p<0.0001

The influence of RNF145 on HCC cell migration was further investigated. After RNF145 was knockout in Hep3B and Huh7 cells, scratch assay was carried out, and scratched cells were photographed at 0 h and 24 h (Fig. 9A). After quantifying, we found that RNF145-knockout HCC cells presented remarkably lower migration rate (Fig. 9B). Altogether, RNF145 loss can attenuate migration of HCC cells, and RNF145 might be required for HCC cell migration.

## Discussion

Owing to the heterogeneity and complexity of HCC, most patients are diagnosed at advanced stages [25]. The non-surgical therapeutic options are often restricted by primary pathophysiological factors determining the tumor microenvironment, especially hypoxia [26]. Hypoxia is a key feature of HCC microenvironment, which is frequent in all stages of HCC progression [27]. Based upon hypoxia genes, the present research classified HCC patients into two hypoxia subtypes, revealing the intratumoral heterogeneity of HCC.

Two hypoxia subtypes presented unique clinicopathologic features, with older age, lower proportion of males, higher proportions of advanced pathologic stage and T stage, in cluster2. In addition, cluster2 exhibited poorer OS, DFS, DSS and PFS outcomes. Most hypoxia genes were up-regulated in cluster2 relative to cluster1, explaining the contribution of hypoxia to HCC progression. The heterogeneity in tumor infiltrating immune cells was also observed between hypoxia subtypes. M0 macrophages, neutrophils, T cells CD8, T cells follicular helper, and Tregs exhibited the higher infiltration in cluster2, with the lower infiltration of M1 macrophages, mast cells resting, monocytes, NK cells activated, and T cells CD4 memory resting. Two hypoxia subtypes had the heterogeneity in genetic mutation. Particularly, cluster1 displayed the higher mutated frequency of CTNNB1, and KMT2D and the lower mutated frequency of TP53, TSC2, ADCY5, HIVEP1, RB1, ATP10D, FBF1, and MAP 4K5.

The hypoxia-relevant gene signature was proposed with LASSO method, composed of ANLN, CBX2, DLGAP5, FBLN2, FTCD, HMOX1, IGLV1-44, IL33, LCAT, LPCAT1, MKI67, PFN2, RNF145, S100A9, and SPP1. It was predicted that high-risk patients presented worse OS outcomes. Additionally, the signature independently and reliably predicted patient prognosis. The high reproducibility was proven in external cohorts. HIF signaling pathway was primarily enriched in high-risk group, demonstrating the notable association of the hypoxia-relevant risk score with hypoxic environment of HCC. Based upon the risk score, we screened a few possible small molecule agents, comprising Doramapimod_1042, JAK1_8709_1718, AZD2014_1441, SB505124_1194, NU7441_1038, ML323_1629, MK-1775_1179, Lapatinib_1558, Sepantronium bromide_1941, Afatinib_1032, Paclitaxel_1080, WEHI-539_1997, and Wee1 Inhibitor_1046. Immune checkpoint blockade has revolutionized the management of HCC. Nonetheless, predictive biomarkers are of limit. In accordance with high expression of immune checkpoints (CD209, CTLA4, HAVCR2, SIRPA, TNFRSF18, TNFRSF4, and TNFRSF9), high T cell inflamed score, low TIDE score and high TMB score, it was inferred that high-risk patients might respond to immunotherapy. Hence, the hypoxia-relevant risk score was potentially utilized for prediction of immunotherapeutic response. Genetic mutations are processed into neoantigens and presented to T cells via MHC proteins [28]. To escape immune eradication, tumor cells utilize checkpoints that suppress T-cell responses. Higher TMB leads to more neoantigens, increased chances of T-cell recognition, and clinically relevant better effects of immunotherapy. There was remarkable heterogeneity in genetic mutation between low- and high-risk groups. Specially, TP53, ADAM12, FCGBP, DNAH10, ADRA1D, CHST3, NLRP2, PIKFYVE, and SVIL occurred with the higher mutated frequency in high- relative to low-risk patients. In contrast, the mutated frequency of DYNC2H1 was lower in high-risk patients.

Among hypoxia-relevant genes, ANLN is required for tumor growth, and targeting ANLN mitigates tumorigenesis and tumor growth in HCC [29]. Overexpressed ANLN correlates to poor prognosis of HCC patients [30]. CBX2 has been proven as an independent prognostic factor of HCC [31]. CBX2 deficiency attenuates proliferation and enhances apoptosis through phosphorylating YAP in HCC [32]. DLGAP5 loss lowers the proliferation and invasion of HCC cells [33]. A recent study demonstrated that Gasdermin D-mediated release of IL-33 from senescent hepatic stellate cells facilitates obesity-associated HCC [34]. Long et al. constructed a prognostic gene signature through comprising two DNA methylation-driven genes (SPP1 and LCAT) for diagnosis, prediction of prognosis and recurrence for HCC [35]. LPCAT1 may alter phospholipid composition and facilitate HCC progression [36]. MKI67 was found to strongly correlate with microvascular invasion that is a main risk factor for recurrence after surgery in HCC [37]. Limited evidence proves that PFN2 is in relation to HCC prognosis [38]. S100A9 expression can be up-regulated by tumor-infiltrating monocytes/macrophages, thus enhancing HCC cell migration and invasion [39]. Until now, the role of RNF145 in HCC is unexplored. Based upon experimental verification, RNF145 was up-regulated in HCC cells after hypoxia, indicating that RNF145 might correlate with HCC hypoxic environment. RNF145 loss mitigated proliferation and migration, and facilitated apoptosis in HCC cells. This demonstrated that RNF145 might be a possible therapeutic target of HCC.

Considering our well-delineated hypoxia-based classification and prognostic signature, computer algorithms should be able to provide more powerful approaches for clinical management of HCC and other diseases. Recently, novel algorithms have been developed for autonomous image processing of medical images including CT and MRI [40–42]. Combined with patient oriented processing approaches [43], the classification and prognostic signature newly discovered by bioinformatic analysis can be more and more helpful for both clinicians and radiologists in the diagnosis, even the establishment of treating plans for individual patients.

The limitations of our research should be acknowledged. Given that all the data for our analysis is collected from public database, more work should be done to validate this retrospective study. For instance, the hypoxia subtypes and hypoxia-relevant prognostic model will be verified in large prospective cohorts, thus facilitating the clinical application. Among hypoxia-relevant genes, we only experimentally validated the role of RNF145 in HCC progression. More experiments will be presented on other hypoxia-relevant genes in HCC.

## Conclusion

Collectively, the hypoxia-based classification and prognostic signature were established, which might assist prognostication and individualized clinical management of HCC patients. In addition, RNF145 was proven as a possible therapeutic target of HCC.

## Supplementary Information

**Additional file 1:** **Supplementary Table 1.** Univariate-cox regression results of 226 hypoxia-relevant genes as protective factors of HCC patients.

**Additional file 2:** **Supplementary Table 2.** Univariate-cox regression results of 258 hypoxia-relevant genes as risk factors of HCC patients.

## Data Availability

The original contributions presented in the study are included in the article/Supplementary Material, further inquiries can be directed to the corresponding author.
