# Supplementary material for: Hypoxia-based classification and prognostic signature for clinical management of hepatocellular carcinoma
Source: World J Surg Oncol. 2023 Jul 22;21:216. doi: 10.1186/s12957-023-03090-x (PMC10362578; doi:10.1186/s12957-023-03090-x)
Supplement: Supplementary file 1 — Additional file 1: Supplementary Table 1. Univariate-cox regression results of 226 hypoxia-relevant genes as protective factors of HCC patients. [file 12957_2023_3090_MOESM1_ESM.docx]

| Gene | HR | P |
| --- | --- | --- |
| MGMT | 0.69006 | 1.00E-04 |
| ALDH2 | 0.70621 | 6.20E-05 |
| HAGH | 0.7352 | 0.00063 |
| GCDH | 0.74354 | 0.00254 |
| ADI1 | 0.76447 | 0.0036 |
| DHRS1 | 0.77001 | 0.00348 |
| RNASE4 | 0.77141 | 0.00061 |
| ECI2 | 0.77343 | 0.02005 |
| GRHPR | 0.77452 | 0.00829 |
| RCL1 | 0.78499 | 0.00354 |
| HSD17B8 | 0.78719 | 0.00949 |
| METTL7A | 0.79193 | 0.00558 |
| FAXDC2 | 0.79394 | 0.00077 |
| ALAS1 | 0.79424 | 0.00314 |
| LCAT | 0.79713 | 1.00E-05 |
| LDHD | 0.79894 | 0.00014 |
| ALDH5A1 | 0.80093 | 0.00392 |
| ALAD | 0.80317 | 0.00544 |
| CLYBL | 0.80967 | 0.01723 |
| ACBD4 | 0.80972 | 0.01734 |
| CRYL1 | 0.82395 | 0.01921 |
| BDH1 | 0.82888 | 0.00019 |
| ECM2 | 0.82905 | 0.00092 |
| CAT | 0.83678 | 0.0278 |
| RIDA | 0.83911 | 0.00662 |
| GSTZ1 | 0.83936 | 0.02029 |
| RAB11B-AS1 | 0.83949 | 0.04675 |
| GADD45G | 0.84162 | 0.00291 |
| CYP27A1 | 0.84542 | 0.00221 |
| ANG | 0.84603 | 0.00166 |
| MLXIPL | 0.85046 | 0.01147 |
| F11 | 0.85109 | 0.00036 |
| SORD | 0.85186 | 0.00384 |
| KLKB1 | 0.85381 | 0.00545 |
| SARDH | 0.85497 | 0.00958 |
| HAAO | 0.85611 | 0.02605 |
| GAMT | 0.85814 | 0.03534 |
| APOC1 | 0.85946 | 0.00213 |
| SLC16A11 | 0.86043 | 0.00033 |
| LRRC3 | 0.86096 | 0.00523 |
| GPT | 0.86173 | 0.0023 |
| RGN | 0.86388 | 0.0024 |
| FTCD | 0.86497 | 6.50E-06 |
| SLC25A15 | 0.86584 | 0.00478 |
| SHMT1 | 0.86731 | 0.02158 |
| RBP4 | 0.86739 | 0.0007 |
| CFB | 0.86843 | 0.01853 |
| CD14 | 0.86849 | 0.04112 |
| RORC | 0.86864 | 0.00132 |
| DMGDH | 0.8703 | 0.0016 |
| ADHFE1 | 0.87268 | 0.01551 |
| FMO4 | 0.87494 | 0.02574 |
| EHHADH | 0.87566 | 0.01113 |
| SLC27A5 | 0.8758 | 0.00095 |
| RP11-116D2.1 | 0.87633 | 0.00103 |
| SEC14L2 | 0.87644 | 0.00346 |
| CPB2 | 0.87658 | 0.0019 |
| CHAD | 0.87725 | 0.00924 |
| EPHX2 | 0.87766 | 0.04323 |
| ANXA10 | 0.87883 | 0.00017 |
| ACOT12 | 0.87917 | 0.00013 |
| IL33 | 0.88023 | 0.019 |
| HPX | 0.88041 | 0.00027 |
| SLCO2B1 | 0.8807 | 0.04724 |
| PDK4 | 0.88169 | 0.00938 |
| DBH-AS1 | 0.88184 | 0.04721 |
| MST1 | 0.88194 | 0.03447 |
| PON3 | 0.88242 | 0.01243 |
| OGDHL | 0.88243 | 0.00047 |
| C6 | 0.88301 | 0.00137 |
| GPT2 | 0.88361 | 0.01948 |
| CFHR4 | 0.88382 | 3.60E-05 |
| ALDH6A1 | 0.88468 | 0.04242 |
| PCK2 | 0.88471 | 0.0455 |
| ABAT | 0.88594 | 0.0128 |
| SEPTIN4 | 0.88709 | 0.04722 |
| PON1 | 0.88709 | 8.10E-05 |
| TMPRSS6 | 0.88729 | 0.00192 |
| ELFN1 | 0.88752 | 0.0016 |
| ACKR2 | 0.88779 | 0.01317 |
| DEPDC7 | 0.88836 | 0.02714 |
| HMGCS2 | 0.8896 | 0.00116 |
| DCXR | 0.88967 | 0.04022 |
| SLC2A2 | 0.88973 | 0.00037 |
| GLYATL1 | 0.89041 | 0.00095 |
| CYP2C9 | 0.89116 | 9.60E-05 |
| AZGP1P1 | 0.89118 | 0.00526 |
| PROC | 0.89148 | 0.02178 |
| SLC6A1 | 0.89184 | 0.00279 |
| FBP1 | 0.89188 | 0.01075 |
| CFHR3 | 0.89266 | 0.00017 |
| A1BG | 0.89338 | 0.00094 |
| CYP3A5 | 0.89438 | 0.00778 |
| CLDN14 | 0.89458 | 0.01497 |
| RNU1-70P | 0.89496 | 0.00182 |
| AMDHD1 | 0.89553 | 0.02196 |
| MTND4P20 | 0.89557 | 0.00049 |
| AMBP | 0.89596 | 0.00961 |
| ITIH1 | 0.8963 | 0.00405 |
| RDH16 | 0.89643 | 0.00054 |
| IGSF23 | 0.89741 | 0.00359 |
| APBA1 | 0.89747 | 0.03533 |
| RBP5 | 0.89774 | 0.03282 |
| G6PC | 0.8978 | 0.00053 |
| BAAT | 0.89915 | 0.01254 |
| KNG1 | 0.90002 | 0.0026 |
| PIPOX | 0.90041 | 0.01986 |
| RAMP1 | 0.90066 | 0.01695 |
| MYRIP | 0.90068 | 0.00241 |
| ADH4 | 0.90148 | 5.00E-06 |
| SPARCL1 | 0.9019 | 0.04364 |
| ADH1B | 0.90224 | 0.00106 |
| AKR7A3 | 0.90232 | 0.00347 |
| CYP4F12 | 0.90282 | 0.0469 |
| AOC4P | 0.90314 | 0.01324 |
| C8A | 0.90325 | 0.00885 |
| HAO1 | 0.90439 | 0.00546 |
| AR | 0.90447 | 0.00894 |
| GPLD1 | 0.90515 | 0.00467 |
| CCDC170 | 0.90551 | 0.03694 |
| AFM | 0.90558 | 0.00112 |
| TFR2 | 0.90607 | 0.02276 |
| FMO3 | 0.90631 | 0.00342 |
| IYD | 0.90639 | 0.00479 |
| ABCA6 | 0.90654 | 0.02357 |
| F13B | 0.90784 | 0.00575 |
| LEAP2 | 0.90875 | 0.03482 |
| PROZ | 0.9091 | 0.01187 |
| MASP2 | 0.90912 | 0.00285 |
| TM6SF2 | 0.90936 | 0.04827 |
| MAT1A | 0.9098 | 0.02289 |
| ADH1A | 0.90985 | 0.00435 |
| FBLN2 | 0.91002 | 0.01498 |
| USH2A | 0.91008 | 0.024 |
| MIR621 | 0.91008 | 0.01655 |
| SLC38A3 | 0.9101 | 0.03357 |
| ACSL6 | 0.91042 | 0.01958 |
| SLC51A | 0.9109 | 0.02021 |
| SLC46A3 | 0.91091 | 0.03347 |
| LPA | 0.91118 | 0.00827 |
| SLC22A1 | 0.9112 | 0.00046 |
| CTH | 0.91122 | 0.02937 |
| UPB1 | 0.9113 | 0.0045 |
| SPP2 | 0.91131 | 0.00021 |
| SLC10A1 | 0.91189 | 0.00023 |
| MAMDC4 | 0.91199 | 0.03796 |
| HSD17B6 | 0.91211 | 0.00588 |
| ANO1 | 0.91219 | 0.02112 |
| TAT | 0.91234 | 0.0007 |
| AZGP1 | 0.91248 | 0.02755 |
| GNMT | 0.91305 | 0.00698 |
| ZNF385B | 0.91313 | 0.01634 |
| C4BPB | 0.91325 | 0.03947 |
| SRD5A2 | 0.91326 | 0.00525 |
| PLG | 0.91331 | 0.01087 |
| RTP3 | 0.91337 | 0.00102 |
| AGXT | 0.91351 | 0.00547 |
| C4BPA | 0.91447 | 0.00882 |
| PAH | 0.9157 | 0.02054 |
| C3P1 | 0.91669 | 0.00374 |
| LECT2 | 0.91802 | 0.00038 |
| CDO1 | 0.91833 | 0.01257 |
| LINC01554 | 0.91866 | 0.00088 |
| SAA4 | 0.91884 | 0.01438 |
| CPS1 | 0.91926 | 0.00052 |
| ADH1C | 0.91953 | 0.00156 |
| APOC3 | 0.91975 | 0.01026 |
| NR1I2 | 0.91991 | 0.00434 |
| ACSM2A | 0.92006 | 0.01169 |
| CA5A | 0.92021 | 0.01526 |
| ALDOB | 0.92043 | 0.00738 |
| AASS | 0.92056 | 0.03908 |
| SLC38A4 | 0.92095 | 0.02517 |
| F12 | 0.92226 | 0.02639 |
| BHMT | 0.92273 | 0.00689 |
| AKR1D1 | 0.92375 | 0.00523 |
| TTR | 0.92407 | 0.0205 |
| AQP9 | 0.92476 | 0.0082 |
| TMEM82 | 0.92541 | 0.03208 |
| APOA1 | 0.9262 | 0.01218 |
| ACE2 | 0.92622 | 0.01288 |
| TTC36 | 0.92755 | 0.0069 |
| APOF | 0.92798 | 0.01558 |
| CYP7A1 | 0.92803 | 0.0013 |
| HULC | 0.92853 | 0.03629 |
| SULT2A1 | 0.92864 | 0.00899 |
| CPN2 | 0.92887 | 0.04143 |
| SLC22A10 | 0.92917 | 0.03397 |
| ABCA8 | 0.92956 | 0.04889 |
| SERPINC1 | 0.92985 | 0.01588 |
| AOX1 | 0.92993 | 0.03144 |
| HPR | 0.93024 | 0.03264 |
| CYP2C8 | 0.93037 | 0.01909 |
| GFRA1 | 0.93062 | 0.01581 |
| CYP2D6 | 0.93067 | 0.04685 |
| SLC17A2 | 0.93125 | 0.02325 |
| CUX2 | 0.93127 | 0.02623 |
| SLC1A2 | 0.93128 | 0.01658 |
| APOA5 | 0.93146 | 0.03907 |
| CYP4A11 | 0.93201 | 0.0429 |
| HJV | 0.93331 | 0.03111 |
| HRG | 0.93333 | 0.00526 |
| ETNPPL | 0.93445 | 0.02949 |
| FETUB | 0.93478 | 0.02768 |
| IGLV2-11 | 0.93574 | 0.04385 |
| LINC01018 | 0.93628 | 0.0032 |
| FAM99A | 0.93637 | 0.01079 |
| GYS2 | 0.93765 | 0.01675 |
| JCHAIN | 0.93791 | 0.04477 |
| UROC1 | 0.93839 | 0.01403 |
| IGLV1-44 | 0.93846 | 0.0333 |
| IGLV3-19 | 0.94007 | 0.02944 |
| IGHV4-59 | 0.94071 | 0.0387 |
| MTRNR2L1 | 0.94096 | 0.01903 |
| OTC | 0.94116 | 0.03778 |
| ACADL | 0.94137 | 0.03716 |
| CFHR5 | 0.94193 | 0.02603 |
| HPD | 0.94245 | 0.02707 |
| HSD17B13 | 0.94264 | 0.00715 |
| F9 | 0.94527 | 0.03749 |
| FAM83A-AS1 | 0.94536 | 0.03574 |
| CYP8B1 | 0.94591 | 0.01672 |
| HGFAC | 0.94658 | 0.01397 |
| HAO2 | 0.94783 | 0.04956 |
| CYP3A4 | 0.95088 | 0.01531 |
| CYP2A6 | 0.95501 | 0.03597 |
