# Supplementary material for: Hypoxia-based classification and prognostic signature for clinical management of hepatocellular carcinoma
Source: World J Surg Oncol. 2023 Jul 22;21:216. doi: 10.1186/s12957-023-03090-x (PMC10362578; doi:10.1186/s12957-023-03090-x)
Supplement: Supplementary file 2 — Additional file 2: Supplementary Table 2. Univariate-cox regression results of 258 hypoxia-relevant genes as risk factors of HCC patients. [file 12957_2023_3090_MOESM2_ESM.docx]

| Gene | HR | P |
| --- | --- | --- |
| CXCL6 | 1.05058 | 0.04107 |
| CAPN6 | 1.05475 | 0.0468 |
| NPTX2 | 1.05504 | 0.04458 |
| TMC5 | 1.05826 | 0.03747 |
| DNER | 1.0609 | 0.04294 |
| C6orf223 | 1.06224 | 0.01707 |
| COL11A1 | 1.06406 | 0.0412 |
| PTGES | 1.06595 | 0.04366 |
| CTAG2 | 1.06799 | 0.00364 |
| PAQR5 | 1.06898 | 0.04088 |
| AGR2 | 1.06967 | 0.00152 |
| LYPD1 | 1.06977 | 0.04876 |
| MEP1A | 1.07028 | 0.00824 |
| PLPP2 | 1.07041 | 0.01543 |
| SLC1A7 | 1.0706 | 0.03743 |
| ARFGEF3 | 1.07352 | 0.03552 |
| PAEP | 1.07624 | 0.00121 |
| CTSE | 1.0763 | 0.01276 |
| CLGN | 1.07747 | 0.03265 |
| PTHLH | 1.07776 | 0.03664 |
| KRT20 | 1.07842 | 0.00418 |
| MMP7 | 1.07882 | 0.00238 |
| CXCL1 | 1.07901 | 0.00592 |
| VLDLR | 1.08145 | 0.02959 |
| SPHK1 | 1.08194 | 0.02648 |
| BACE2 | 1.08236 | 0.04607 |
| INAVA | 1.0832 | 0.0106 |
| CXCL5 | 1.08345 | 0.00145 |
| SLC6A8 | 1.08419 | 0.03548 |
| LINC00665 | 1.08426 | 0.04519 |
| HKDC1 | 1.08571 | 0.0316 |
| EPS8L3 | 1.08852 | 0.00966 |
| SERPINE2 | 1.08958 | 0.04846 |
| CA9 | 1.09031 | 0.0002 |
| CREB3L1 | 1.0904 | 0.03883 |
| CDCA7 | 1.09066 | 0.02187 |
| PYCR1 | 1.09132 | 0.01176 |
| UCHL1 | 1.09206 | 0.00301 |
| GCNT3 | 1.09257 | 0.00596 |
| MMP9 | 1.09357 | 0.01918 |
| PNCK | 1.09358 | 0.00069 |
| CD24 | 1.09361 | 0.00871 |
| SLC6A6 | 1.09414 | 0.04935 |
| SSTR2 | 1.09596 | 0.02619 |
| APLP1 | 1.09698 | 0.01234 |
| PTK7 | 1.09777 | 0.03939 |
| NEURL3 | 1.09837 | 0.01832 |
| C12orf75 | 1.09905 | 0.01742 |
| GRAMD1B | 1.10179 | 0.01183 |
| CDKN1C | 1.10277 | 0.0459 |
| HTRA3 | 1.10534 | 0.01672 |
| TUSC3 | 1.10546 | 0.01486 |
| B3GNT5 | 1.10571 | 0.02584 |
| NXPH4 | 1.10611 | 0.0032 |
| S100A8 | 1.10642 | 0.03409 |
| SERPINE1 | 1.10818 | 0.0238 |
| FABP3 | 1.10913 | 0.02652 |
| PGAP4 | 1.11042 | 0.0133 |
| TMEM45A | 1.11225 | 0.00791 |
| GLDN | 1.11271 | 0.00107 |
| NRSN2 | 1.1131 | 0.02465 |
| ITGA2 | 1.11324 | 0.03149 |
| MTCL1 | 1.11339 | 0.01413 |
| CXCL8 | 1.11354 | 0.00074 |
| PLTP | 1.11477 | 0.0324 |
| MSC | 1.11599 | 0.00143 |
| NCEH1 | 1.11633 | 0.04829 |
| TMEM132A | 1.11776 | 0.01889 |
| DNM1 | 1.11855 | 0.01486 |
| S100A6 | 1.11891 | 0.01669 |
| OXCT1 | 1.12109 | 0.01363 |
| AKR1B1 | 1.1211 | 0.04997 |
| SLC12A2 | 1.12155 | 0.04729 |
| ZNF320 | 1.12157 | 0.01612 |
| HK2 | 1.12343 | 0.00329 |
| SPP1 | 1.12347 | 4.60E-06 |
| TTC39A | 1.12469 | 0.00516 |
| SEMA6A | 1.12691 | 0.01129 |
| ELF4 | 1.12853 | 0.0412 |
| SOX4 | 1.12886 | 0.01823 |
| TSPAN15 | 1.12928 | 0.03272 |
| TNFRSF11B | 1.1294 | 0.00177 |
| CDR2L | 1.12969 | 0.04773 |
| ACSS1 | 1.12975 | 0.0325 |
| PDE4A | 1.12985 | 0.04651 |
| CHST11 | 1.13087 | 0.0301 |
| IER5L | 1.13255 | 0.04199 |
| ENO2 | 1.13657 | 0.00634 |
| ICAM1 | 1.13734 | 0.03074 |
| KRT17 | 1.13758 | 0.00047 |
| CTSV | 1.13846 | 0.00015 |
| TMSB10 | 1.13912 | 0.01797 |
| MARVELD1 | 1.13972 | 0.02941 |
| KLHL29 | 1.14058 | 0.00979 |
| PFKFB3 | 1.1407 | 0.00769 |
| CTHRC1 | 1.1422 | 0.00093 |
| PKIB | 1.14259 | 0.00105 |
| FCGBP | 1.14285 | 0.00157 |
| PLA2G7 | 1.14325 | 0.02255 |
| HSPA6 | 1.14426 | 0.00872 |
| FMNL2 | 1.14478 | 0.01574 |
| BAIAP2L2 | 1.14667 | 0.00239 |
| NCK2 | 1.1469 | 0.02041 |
| TREM2 | 1.1469 | 0.00699 |
| CHGA | 1.14719 | 7.30E-05 |
| FZD1 | 1.14788 | 0.01819 |
| TYRO3 | 1.14867 | 0.00092 |
| ASNS | 1.14904 | 0.00377 |
| SLC7A1 | 1.1502 | 0.00433 |
| DBN1 | 1.15021 | 0.01396 |
| RGS2 | 1.1513 | 0.00319 |
| TMEM54 | 1.15155 | 0.00547 |
| MFSD6 | 1.15237 | 0.0371 |
| DSG2 | 1.15291 | 0.00217 |
| PRSS23 | 1.15355 | 0.03555 |
| TNFAIP8 | 1.15451 | 0.03512 |
| LOX | 1.15453 | 0.00583 |
| TNFRSF21 | 1.15627 | 0.0116 |
| NFE2L3 | 1.15697 | 0.01898 |
| TGFB1 | 1.15961 | 0.01595 |
| SINHCAF | 1.16006 | 0.02879 |
| PLCD3 | 1.16277 | 0.00756 |
| ARNTL2 | 1.16314 | 0.00641 |
| NDUFA4L2 | 1.16326 | 0.01201 |
| RASSF8 | 1.16498 | 0.0193 |
| IGFBP3 | 1.16618 | 0.01012 |
| RASA3 | 1.16714 | 0.02014 |
| IER3 | 1.16727 | 0.00363 |
| PFKP | 1.1686 | 0.00052 |
| PLXNA3 | 1.16936 | 0.01977 |
| SERPINI1 | 1.16966 | 0.01519 |
| PAM | 1.17257 | 0.02064 |
| SCRN1 | 1.17324 | 0.00146 |
| SMIM3 | 1.17358 | 0.00615 |
| SLC7A7 | 1.17394 | 0.0158 |
| PLP2 | 1.17559 | 0.00642 |
| EGLN3 | 1.17619 | 3.90E-05 |
| SLC7A8 | 1.17655 | 0.01099 |
| PHLDA2 | 1.17672 | 0.00051 |
| RHPN1 | 1.18122 | 0.00848 |
| RTL6 | 1.18248 | 0.008 |
| STK39 | 1.18335 | 0.00096 |
| ZNF532 | 1.18674 | 0.01256 |
| OSBPL3 | 1.18711 | 0.022 |
| NCS1 | 1.18763 | 0.00278 |
| SLC25A24 | 1.18806 | 0.00095 |
| TRNP1 | 1.18831 | 1.70E-05 |
| MMP14 | 1.18845 | 0.00538 |
| PGF | 1.18901 | 0.00232 |
| PLEKHA2 | 1.18972 | 0.0335 |
| MYLIP | 1.19117 | 0.03202 |
| FAM117B | 1.19218 | 0.02172 |
| KCTD17 | 1.19287 | 0.00077 |
| SERPINH1 | 1.19308 | 0.03104 |
| PLAUR | 1.19321 | 0.00312 |
| SLC38A1 | 1.19376 | 0.0002 |
| IKBKE | 1.19527 | 0.00253 |
| ASRGL1 | 1.19543 | 0.0006 |
| TEAD2 | 1.19552 | 0.00563 |
| PLBD1 | 1.19593 | 0.00018 |
| DEPDC1B | 1.19608 | 0.00012 |
| IGSF3 | 1.19625 | 0.00018 |
| FCER1G | 1.19714 | 0.01104 |
| PKM | 1.19717 | 0.00024 |
| UAP1L1 | 1.19724 | 0.00037 |
| CLSTN1 | 1.19919 | 0.01353 |
| KIF3C | 1.20119 | 0.0016 |
| S100A11 | 1.20134 | 0.00092 |
| ABCC1 | 1.20175 | 0.00131 |
| HDAC7 | 1.20176 | 0.03882 |
| HMOX1 | 1.20351 | 0.00495 |
| UHRF1 | 1.20435 | 0.00034 |
| CDCA7L | 1.20535 | 0.00347 |
| SFXN3 | 1.20653 | 0.00776 |
| CARMIL1 | 1.20685 | 0.00123 |
| S100A9 | 1.20777 | 5.20E-06 |
| HOMER3 | 1.20991 | 0.00224 |
| PAFAH1B3 | 1.21046 | 0.00112 |
| TOP2A | 1.2133 | 0.00017 |
| RAP1GAP | 1.2152 | 0.0006 |
| OLFML2B | 1.21565 | 0.00166 |
| MYBL2 | 1.21624 | 9.60E-06 |
| UNC5B | 1.21689 | 0.00184 |
| PFN2 | 1.21746 | 3.50E-05 |
| HIF1A | 1.21857 | 0.00786 |
| LAMB1 | 1.22052 | 0.00235 |
| CENPF | 1.22097 | 0.00026 |
| SPRED1 | 1.22123 | 0.01019 |
| FSCN1 | 1.22854 | 0.00483 |
| AURKB | 1.22954 | 0.00024 |
| LHFPL2 | 1.22966 | 0.00245 |
| CHAF1B | 1.2314 | 0.00032 |
| MFSD10 | 1.2317 | 0.00386 |
| RAB3IL1 | 1.23277 | 0.00083 |
| KIF18B | 1.23798 | 3.50E-05 |
| BUB1B | 1.23871 | 9.70E-05 |
| SPRING1 | 1.23965 | 0.00591 |
| ADGRE5 | 1.24209 | 0.00131 |
| FABP5 | 1.2429 | 0.00178 |
| KIF23 | 1.24518 | 6.30E-05 |
| ITGAV | 1.24546 | 0.00145 |
| TOR4A | 1.25136 | 0.00051 |
| UBE2C | 1.25321 | 3.20E-05 |
| KIF4A | 1.25492 | 2.80E-05 |
| NCAPH | 1.25593 | 0.00016 |
| NT5DC2 | 1.25794 | 8.00E-05 |
| STC2 | 1.25927 | 2.80E-05 |
| OGFRL1 | 1.25994 | 0.00673 |
| MMD | 1.26134 | 0.00149 |
| NUF2 | 1.26263 | 2.30E-05 |
| ARHGEF2 | 1.2632 | 0.00262 |
| PIP4P2 | 1.26344 | 0.00016 |
| MKI67 | 1.2641 | 4.10E-05 |
| TTYH3 | 1.26555 | 0.00181 |
| FAM102B | 1.2699 | 0.00205 |
| SLC16A3 | 1.27007 | 1.40E-06 |
| GTSE1 | 1.27052 | 2.20E-05 |
| LPCAT4 | 1.27109 | 0.00074 |
| WDR62 | 1.27257 | 0.00012 |
| BCAT1 | 1.27296 | 5.80E-05 |
| NDRG1 | 1.27698 | 0.00013 |
| PDE7A | 1.27776 | 0.00334 |
| CSF1 | 1.27961 | 0.00144 |
| DAB2 | 1.28361 | 0.00018 |
| LIMK1 | 1.28899 | 0.00086 |
| SLC1A5 | 1.29173 | 3.50E-07 |
| LAPTM4B | 1.29261 | 6.40E-05 |
| BUB1 | 1.29272 | 1.50E-05 |
| AGRN | 1.29629 | 0.00046 |
| ECT2 | 1.29802 | 2.00E-05 |
| ANLN | 1.30001 | 2.20E-06 |
| ADAM9 | 1.3002 | 0.00011 |
| PNMA1 | 1.30157 | 0.00064 |
| SMOX | 1.3082 | 1.90E-05 |
| SLC2A1 | 1.30924 | 6.40E-07 |
| CDC20 | 1.3093 | 3.20E-07 |
| KIF11 | 1.31307 | 3.60E-05 |
| PLK1 | 1.31473 | 1.90E-06 |
| PLXNA1 | 1.31512 | 0.00019 |
| DLGAP5 | 1.31855 | 1.80E-06 |
| MEX3A | 1.32672 | 2.60E-06 |
| IMPDH1 | 1.33182 | 2.30E-06 |
| ALDOA | 1.33669 | 4.80E-05 |
| KIF2C | 1.34329 | 2.30E-07 |
| KIF20A | 1.34738 | 1.00E-06 |
| PRR11 | 1.34775 | 1.30E-06 |
| TRIP13 | 1.35644 | 1.70E-07 |
| G6PD | 1.36009 | 4.60E-09 |
| HILPDA | 1.36423 | 1.80E-07 |
| STX3 | 1.37779 | 0.00011 |
| RNF24 | 1.38446 | 9.80E-05 |
| CBX2 | 1.385 | 2.90E-08 |
| LPCAT1 | 1.39799 | 1.60E-07 |
| CDCA8 | 1.41771 | 7.90E-08 |
| GAS2L3 | 1.42261 | 1.70E-07 |
| LMNB2 | 1.47138 | 6.20E-06 |
| SPINDOC | 1.4882 | 2.50E-06 |
| RNF145 | 1.51382 | 4.90E-06 |
